# Serbian KINDL questionnaire for quality of life assessments in healthy children and adolescents: reproducibility and construct validity

**DOI:** 10.1186/1477-7525-7-79

**Published:** 2009-08-28

**Authors:** Dejan Stevanovic

**Affiliations:** 1 Department of Psychiatry, General Hospital Sombor, Apatinski put 38, 25000 Sombor, Serbia

## Abstract

**Background:**

The KINDL questionnaire is frequently used to evaluate quality of life (QOL) and the impacts of health conditions on children's everyday living. The objectives of this study were to assess the reproducibility and construct validity of the Serbian KINDL for QOL assessments in healthy children and adolescents.

**Methods:**

Five hundred and sixty-four healthy children and adolescents completed the KINDL. Reproducibility was analyzed using the intraclass correlation coefficient (ICC). Confirmatory factor analysis (CFA) was performed to assess the structure of the KINDL - construct validity.

**Results:**

The intraclass correlation coefficients ranged from 0.03 to 0.84 for the subscales and total score. A second order CFA model as originally hypothesized was tested: items (24), primary factors (six subscales), and one secondary factor (QOL). The fit indexes derived from a CFA failed to yield appropriate fit between the data and the hypothesized model.

**Conclusion:**

Majority of the subscales and total KINDL possess appropriate reproducibility for group comparisons. However, a CFA failed to confirm the structure of the original measurement model, indicating that the Serbian version should be revised before wider use for QOL assessments in healthy children and adolescent.

## Background

Nowadays, when quality of life (QOL) has become a universally accepted concept for measuring the impact of different aspects of life on general well-being and everyday functioning, important perspectives are placed on the cross-cultural settings. The cross-cultural settings of QOL represent integral parts in the labelling, promotion and drug regulatory process, public health reporting, epidemiological researches, and multinational clinical trails [1-3]. However, appropriate QOL measures should be available across different cultures that could be used for such purposes. This implies that QOL measures need to be simultaneously developed across different cultures, respecting cultural diversities of each, or to be translated and validated form ones into other languages ensuring measurement equivalence between the original and new versions, but respecting the cultural distinctions of the new ones.

The KINDL, a generic questionnaire for measuring QOL in children and adolescents, is frequently used in Germany and abroad to evaluate the impacts of health conditions on children's everyday living [4,5]. This measure considers QOL as a psychological construct including physical, psychosocial, and functional aspects of well-being and daily functioning [4]. Moreover, it possesses a well-validated measurement model with items grouped in six subscales that assess the main components of children and adolescents QOL and well-being. This structure allows it to be used for QOL assessments in divers groups of healthy children and adolescents, but also for quality of life assessments related to a particular health condition. An extensive research showed the KINDL is an appropriate questionnaire for QOL assessments with satisfactory measurement properties [6]. Over the years, it was translated and adapted into several languages and the validation studies reported the translated versions could provide reliable and valid measurements as the original and could be used in pediatric cross-cultural comparisons [7-13].

For the Serbian version, several validation steps were planned in order to achieve appropriate measurement properties and to claim the translation is equivalent to the original. Two were already undertaken - a translation-adaptation and basic psychometric study, where the content and basic measurement properties were analyzed in a healthy population [13]. It was reported that the Serbian translation possesses relevant QOL domains, good feasibility and acceptability, and it could provide reliable assessments for group comparisons. The next validation steps are to analyze stability of the translation in repeated assessments and to explore its hypothesized theoretical model in healthy children and adolescents. Simultaneously, we evaluate the measurement properties of the KINDL in different pediatric populations to fulfill the paramount aim of developing a standardized measure for QOL assessments in Serbia, where so far there has been none.

Therefore, this study was organized with the aims to assess the reproducibility and construct validity of the Serbian KINDL for QOL assessments in healthy children and adolescents. Considering that we already have the hypothesized theoretical model of the KINDL [4], confirmatory factory analysis was used to study construct validity.

## Methods

### Sample

School psychologists contacted 800 pupils (aged 8-16 years and equally boys and girls) from nine elementary schools in Western Vojvodina to participate in the study. They informed all children and adolescents about the purpose of the study, as well as their parents and teachers. Those agreed to participate and returned the written consent from the parents completed the questionnaire in the schools to prevent a low responding rate. The participants were instructed carefully how to fill the KINDL out. One hundred and twenty randomly selected pupils completed the questionnaires after a seven-day period.

The data from healthy subjects were used for the present analysis and those with major psychological or physical chronic diseases or acutely diseased were not considered relevant. As in the previous study, only health subjects were included, assuming to develop a questionnaire with appropriate measurement properties for QOL assessments in healthy populations [13]. The data about the subjects' health were taken from medical records available in schools.

### Questionnaire

The Serbian Kid-KINDL (8-12 years) and the Kiddo version (13-16 years) are self-report questionnaires developed in the previous study [13]. Each version contains 24 Likert-scaled items in six general subscales: Physical well-being - PW, Emotional well-being - EW, Self-esteem - SE, Family - FAM, Friends - FRI, and School - SC. The score of each item ranges from 1 (never) to 5 (always), while the total of the subscales and overall raw score are formed from the items' means. The raw score are transformed into a 0-100 scale, with higher scores indicating better QOL. The questionnaires and the scoring procedures are provided at the official website [5].

### Statistical analysis

The distribution of missing data was calculated as the percentage of missing responses on all possible responses. Only subscales with less than 30% of missing items were considered, whereby mean value replacement dealt with such missing values. Mean (M) and standard deviation (SD) was calculated for each item, subscale, and total.

Reproducibility, test-retest reliability, concerns the degree to witch repeated assessments in stable persons produce similar responses [3]. It was evaluated using the intarclass correlation coefficient - ICC, the two-way random method of absolute agreement [3]. Assuming reliability is the degree to which people can be distinguished from each other, the KINDL's ICCs should be 0.6 or higher for healthy group comparisons. The retest took place seven days latter.

Construct validity was assessed using factor analysis that combines observable variables into unobservable, latent variables, giving insights into the theoretical model of some construct [3,14]. This is known as factorial validity that is assessed using explorative factor analysis (EFA) and/or confirmative factor analysis (CFA). The present study gave priority to CFA, whereas we already have the hypothesized theoretical model of the KINDL assuming to be confirmed as valid for QOL assessments and it is not necessary to re-explore the latent variables using EFA. Moreover, the current perspectives are to use CFA in QOL models, whereas EFA could produce strange combinations of QOL items with unexpected latent constructs [3]. This is mainly because QOL questionnaires often combine items with a causal relationship with the latent variables, causal variables, and items dependant upon the latent variables, indicator variables, while EFA requires only the later [3,15,16]. Finally, CFA provides some data on convergent (the extent to which similar theoretical constructs are related) and discriminant validity (the extent to which different theoretical constructs are relatively unrelated) as the aspects of construct validity [14].

A CFA was conducted using Analysis of Moment Structures Version 7 (AMOS-7) on a model representing the items and the corresponding factors as originally assumed. Therefore, the tested model, as a second order CFA model, had three levels: items (24), primary factors (six subscales), and one secondary factor (QOL). The primary goal is to determine the goodness of fit between the hypothesized model and the sample data. To test the hypothesized model the variance-covariance matrix was used and maximum likelihood (ML) estimation was employed. ML is robust in terms of using non-continuous data and there is evidence of robustness in the terms of the violation of multivariate normality assumption [17,18]. However, Bollen-Stine bootstrap and associated test of overall model fit were used to study and manage the effects non-normality in the underlying database since research has also demonstrated that ML test statistic (TML) and ML parameter standard errors may be affected when data deviate form normal [17,18]. Bollen-Stine bootstrap provides more realistic standard errors if there is serious departure from multivariate normality. Based on the recommendations, 2,000 bootstrap samples were drawn to obtain overall model fit and 250 bootstrap samples to obtain parameter estimates and associated standard errors [17]. Model identification was established by estimating the factor variances and fixing one factor loading to 1.0 for each factor. The following statistics assessed the adequacy of the model, indirectly construct validity, as the degree of fit between estimated and observed variance: chi square, Tucker Lewis Index (TLI) (>0.90 acceptable, >0.95 excellent), the Comparative Fit Index (CFI) (>0.90 acceptable, >0.95 excellent), and root mean square error of approximation (RMSEA) (<0.08 acceptable, <0.05 excellent) [16-19]. It was assumed the factor loadings of the items within the subscale and the standardized coefficient of the subscales should be at least moderate to support convergent validity, while the correlations between the estimated parameters of the latent factors should be low to support discriminant validity [3,18,20].

## Results

The overall responding rate was 80% for the children and 77% for the adolescents, while the amounts of missing data were 0.17% and 0.32%, respectfully. The Kid completed 303 subjects (160 males and 143 females, mean age 10.77 ± 1.25 years) and the Kiddo 261 (114 males and 147 females, mean age 14.02 ± 0.84).

The reproducibility of majority of the subscales was above 0.6 and appropriate (Table 1). For the total score, the ICC was above 0.8. However, some subscales, like the School Kiddo with the ICC of 0.03, possess very low levels of reproducibility.

**Table 1:** Means (M), standard deviations (SD), and the intraclass correlation coefficients (ICC) of the KINDL questionnaires

KINDL	Kid		Kiddo	
Subscale	M (SD)	ICC n = 63	M (SD)	ICC n = 33

Physical well-being	4.07 (0.66)	0.55	4.03 (0.65)	0.63

Emotional well-being	4.29 (0.58)	0.64	4.141 (0.55)	0.51

Self-esteem	3.87 (0.75)	0.6	3.87 (0.74)	0.75

Family	4.41 (0.55)	0.57	4.52 (0.57)	0.66

Friends	4.07 (0.66)	0.7	4.18 (0.68)	0.54

School	3.61 (0.81)	0.62	3.13 (0.79)	0.03

Total QOL score	4.05 (0.45)	0.84	4.02 (0.43)	0.8

**Figure 1:**
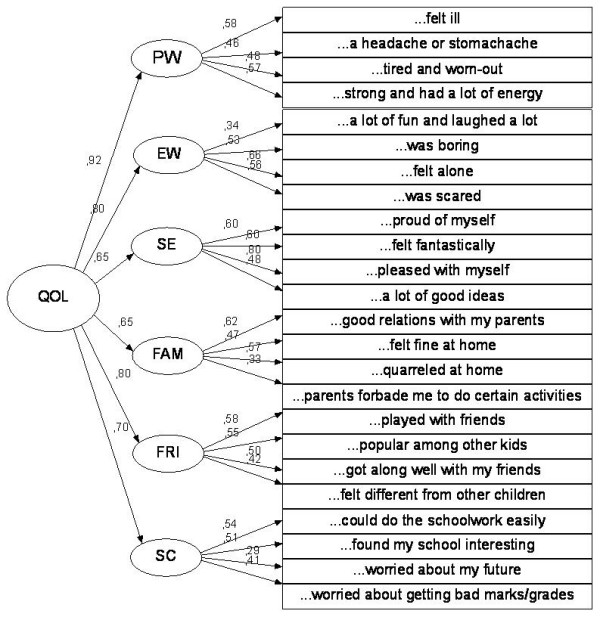
**Final second-ordered CFA model for the Kid-KINDL**. Physical well-being - PW, Emotional well-being - EW, Self-esteem - SE, Family - FAM, Friends - FRI, and School - SC.

**Figure 2:**
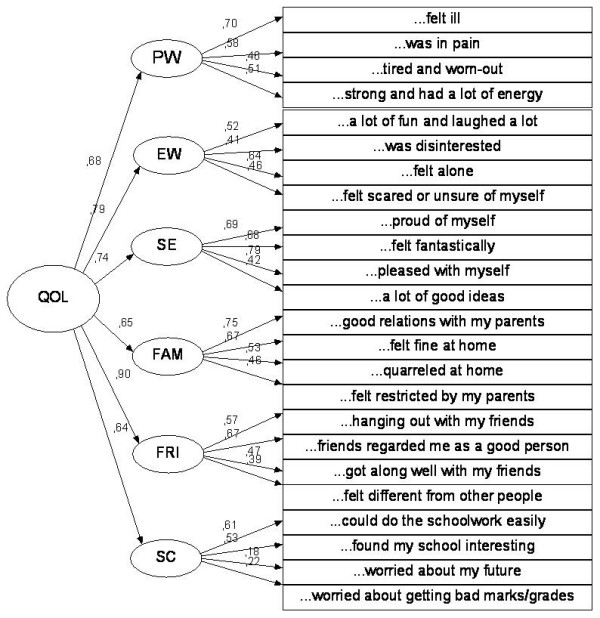
**Final second-ordered CFA model for the Kiddo-KINDL**. Physical well-being - PW, Emotional well-being - EW, Self-esteem - SE, Family - FAM, Friends - FRI, and School - SC.

The final second-order CFA models for both versions are presented in Figure 1 and 2. Above the arrows pointed at the observable variables (rectangles) are given their factor loadings (standardized parameters) and the standardized regression weights of the subscales on the total score are given on the left side of the figures.

The fit indices indicated a bad fit of the data to the hypothesized structure. For the Kid-KINDL, the average chi-square from the 2000 bootstrap samples was 316.38 (SE = 1.05), with Bollen-Stine bootstrap p = .000, while TLI = 0.67, CFI = 0.706, and RMSEA = 0.077. For the Kiddo-KINDL, the average chi-square from the 2000 bootstrap samples was 325.21 (SE = 1.17), with Bollen-Stine bootstrap p = .000, while TLI = 0.618, CFI = 0.66, and RMSEA = 0.092.

The factor loadings varied within each subscale of both versions from low (0.18) to moderate/high (0.79) indicating different level of associations between the latent factors and the respective items (Figure 1 and 2). On the other hand, the correlations between the factors were very low ranging 0.05-0.09 (details not given).

Finally, the standardized coefficient values are moderate (0.64) to high (0.92) for the subscales.

## Discussion

This study further assessed the measurement properties of the Serbian KINDL questionnaire for QOL assessments in healthy children and adolescents. Here, the results reported the translation has appropriate stability in repeated assessments for general groups' comparisons, but the hypothesized theoretical model of QOL is not appropriately represented with the KINDL items.

The reproducibility, as test-retest reliability, of the Serbian KINDL is different across the subscales, ranging from very low (0.03) to moderate (0.75) and it is high (0.8 and 0.84) for the total score only. The Kid version is more stable in repeated assessments than the Kiddo. This level of measurement stability for some subscales is possible to explain with assumption the concepts measured by the items of that subscales are possibly more dynamic in nature and sensible to even subtle changes in QOL than expected for healthy individuals. Taking into account the results of internal consistency from the previous study, where Cronbach's coefficient ranged 0.42-0.72 for the subscales and 0.8 for the total, the level of reliability indicates the total KINDL could only produce reliable assessments for group comparisons [13]. On the contrary, the subscales could produce reliable measurements only for basic evaluations, like sorting subjects or preliminary decisions, considering that some possess inappropriate reliability as an indicator of low discriminatory ability [3]. These data requires more explorations, whereas the recent researches of the Taiwanese version of the Kiddo-KINDL and the Spanish KINDL in healthy populations also reported very similar levels for test-retest reliability [7,12].

The indices from the CFA analysis show the data failed to fit appropriately the hypothesized model of the KINDL, whereas they were below acceptable ranges [3,18]. This implies the original theoretical model could be discarded for the Serbian version and appropriate construct validity is not possible to support for valid QOL assessments. From this analysis, it was observed that the items share common latent construct partially, whereas there are low to moderate associations between the subscales and the respective items (based on the factor loadings) with a high variability of the associations within each subscale of both versions. On the contrary, the correlations between the factors were very low between the subscales, showing the subscales measure different constructs to a substantial degree. Together, these findings suggest that there is a partial level of convergent validity, while the subscales possess even excellent discriminant validity. Placing these observations on the continuum of construct validity, we have on its very left side an excellent distinctiveness of the KINDL subscales, discriminant validity, and somewhere on its middle a moderate possibility of the items to measure common underlying constructs of each domain. Therefore, the above findings show that there are complex associations among the items and their underlying constructs are incompletely represented with the present subscales, although they had strong effects on the total score, suspecting that there might be some third constructs involved in these relations and it needs to be discovered in the future examinations of construct validity [3,14].

The present study is the only one to use CFA for the KINDL in healthy children and adolescent, so it is hard to compare the findings. Nevertheless, the findings from the studies of exploratory factor analysis performed on healthy samples showed the subscales possess unimportant items or some that could be regrouped differently, suggesting revisions for the KINDL [8,10,13]. For the model studied here, AMOS suggested several modification indices that would let to the model improvement as the means of structural equation modeling [3,20]. However, this is beyond the article's scope and such a revision should be best undertaken applying a cross-cultural simultaneous approach to ensure comparability of different national versions and to avoid running into results due to chance. An important consideration during a revision shall be to study the causal effects of those items that influence QOL, causal variables, separately from those indicating a QOL level, indicator variables [3,16].

The study has some limitations that could explain the results as well. First, restricting the sample to healthy subjects leads to restricted distribution of scores and variances, therefore the results of a CFA might be significantly affected. Further, the results might be also affected even Bollen-Stine bootstrap was used to manage the effect of deviation form normality, so the usage of polychoric correlations would be an alternative. Finally, there is no available QOL measures in Serbia with appropriate measurements characteristics against witch to confirm the results of construct validity and no studies reported evaluating the KINDL with CFA in healthy subjects.

## Conclusion

Two important conclusions are here. First, the Serbian KINDL possesses appropriate reproducibility for group compressions, but priorities should be given to the total score. The subscales should be used with precautions, considering that some of them are not stable in producing reliable results in repeated assessments. Second, a CFA failed to confirm the original model of the KINDL and its six subscales, so its construct validity remained unsupported for valid QOL assessments in healthy children and adolescents.

Based on this and the previous study as well [13], it is be inferred the Serbian KINDL could produce relatively reliable, but insufficiently valid QOL assessments in healthy children and adolescents. Consider these negative findings it is advised to replicate the study to ensure whether the current KINDL measurement model is appropriate or not for QOL assessments in healthy children and adolescents in Serbia. In the meanwhile, the psychometric properties of the translation for QOL assessments in different population with chronic diseases will be reported that would add clearer insights into its measurement properties and direct eventual revisions.

## Abbreviations

KINDL: German questionnaire for measuring quality of life in children and adolescents; QOL: quality of life; CFA: confirmatory factor analysis; TLI: Tucker Lewis index; CFI: comparative fit index; RMSEA: root mean square error of approximation.

## Competing interests

The author declares no financial competing interests. This is the third study about the Serbian KINDL that was translated in cooperation and approved by Prof. Ulrike Ravens-Sieberer.

## Authors' contributions

The entire study was organized and presented by the author.
